# Prevalence, Species Composition and Associated Factors of Ectoparasite Infestations in Dogs in Isfahan, Central Iran

**DOI:** 10.1002/vms3.71191

**Published:** 2026-08-19

**Authors:** Nafas Rafiyan, Farnaz Malekifard, Bijan Esmaeilnejad

**Affiliations:** ^1^ Department of Pathobiology Faculty of Veterinary Medicine Urmia University Urmia Iran

**Keywords:** dog, flea, Isfahan, lice, mite, tick

## Abstract

**Background:**

Infestation of canines with ectoparasites is a significant concern worldwide in both medical and veterinary fields, especially in Iran, due to the transmission of pathogenic agents.

**Objectives:**

This study aimed to assess the prevalence and diversity of ectoparasites found in dogs in Isfahan County.

**Methods:**

A total of 138 dogs—comprising 57 stray dogs, 38 herd dogs and 43 owned dogs—were randomly selected and examined between July 2023 and July 2024. The body surfaces of the dogs were inspected for ectoparasites, including ticks, mites, lice and fleas. These parasites were directly removed from the skin of the examined dogs for identification.

**Results:**

Results showed that among the 138 dogs examined, 54 (39.13%) hosted external parasites. Of these, 26 (48.14%) were affected by ticks, 15 (27.77%) by mites, 9 (16.66%) by fleas and 4(7.40%) by lice. A significant difference in prevalence was observed based on the sex and age of the dogs (*p* < 0.05). The highest incidence of external parasite infestation occurred during the spring season.

**Conclusions:**

These findings indicate a notable prevalence of ectoparasites among dogs in Isfahan County. Given the known zoonotic potential of several identified species, these results highlight the need for further studies on pathogen transmission and strengthened veterinary and public health measures.

## Graphical abstract

Ectoparasite infestation was detected in 39.13% of 138 examined dogs in Isfahan, Iran. Ticks were the most prevalent (57.4%), followed by mites (25.9%), fleas (11.1%) and lice (5.6%). Infestation rates showed significant differences according to sex and age (*p* < 0.05), with the highest prevalence observed in spring.

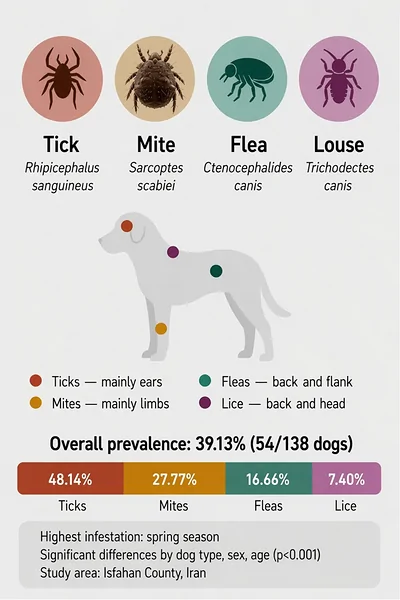

**Abbreviations**
Chi‐square: Chi‐square test
KOH: Potassium hydroxide
Mann: Whitney U test
spp: species plural
SPSS: Statistical Package for the Social Sciences

## 1 Introduction

Ectoparasites, which include ticks, fleas, lice and mites, are major contributors to both pruritic and non‐pruritic skin issues in dogs. These infestations can result in hypersensitivity, anaemia, secondary infections and in severe cases, death, depending on the animal's nutritional status and immune system, as well as the severity of the infestation (Wall and Shearer 2008; Ebrahimzade et al. 2016).

These arthropods not only inflict direct harm on canine hosts but also serve as vectors for various pathogens, transmitting viruses, bacteria, protozoa and helminths to dogs, other animals and humans (Dantas‐Torres and Otranto 2014; Abdulkareem et al. 2019). For example, lice like *Trichodectes canis* can serve as intermediate hosts for cestodes such as *Dipylidium caninum*, which can infect both children and adults, although young children are particularly at risk owing to their closer contact with pets (Scott et al. 2001; Ebrahimzade et al. 2016). Mites like *Sarcoptes scabiei* can lead to sarcoptic mange, a highly contagious zoonotic disease (González et al. 2004; Mosallanejad et al. 2012).

Detection methods for ectoparasites typically include deep and superficial skin scraping, acetate tape preparation, full‐body combing, otic swabs and various clinical trials (Scott et al. 2001). In urban and suburban settings, where dogs are commonly kept as pets, regular health check‐ups play a crucial role in preventing ectoparasite infestations. Understanding the different species, their density and prevalence is essential for effective control measures (Scott et al. 2001; Nuchjangreed and Somprasong 2007).

In areas with large dog populations, such as urban and peri‐urban regions, infestations of ectoparasites persist as a significant public health issue, worsened by factors like insufficient sanitation, lack of veterinary care and environmental conditions that support parasite survival (Xhaxhiu et al. 2009; Mwangengwa and Rhanga 2023).

Previous research has shown a significant occurrence of ectoparasite infestations in dogs across various geographical regions, with differences based on factors like climate, management of hosts and the distribution of parasite species (Durden et al. 2005; Gracia et al. 2008). Recent studies in Iran have highlighted significant seasonal and geographical variation in ectoparasite infestation patterns among livestock and dogs (Shirzadfar et al. 2025; Abbasi 2025a). Findings highlight the endemic problem of ectoparasites in tropical and subtropical regions, where control measures, such as dipping treatments (immersion in acaricide solutions for ectoparasite control), are often compromised by reinfestation from unsensitized environments and inadequate hygiene practices (Beresford‐Jones 1981; Thompson et al. 1993).

Understanding ectoparasite identification and distribution is crucial for developing effective control programs and strategies. However, there is a lack of data regarding ectoparasite prevalence in specific areas of Iran, particularly in urban centres like Isfahan, where close interactions between humans and dogs, along with varying management practices, may increase zoonotic risks (Šlapeta et al. 2011; Bahrami et al. 2012). This study aimed to assess the prevalence, species distribution and associated factors of ectoparasite infestations in dogs in Isfahan, Iran, to guide targeted veterinary actions and public health initiatives.

## 2 Material and Methods

### 2.1 Field Study Area

**FIGURE 1:**
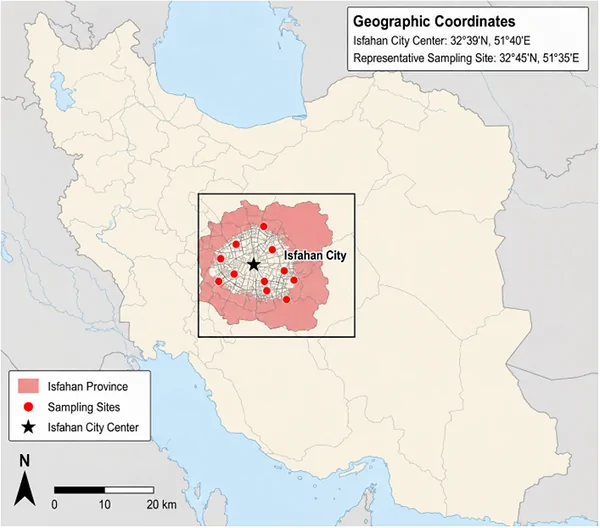
Geographic location of the study area. (A) Map of Iran showing the location of Isfahan Province (highlighted in red). (B) Enlarged map of Isfahan City illustrating the distribution of the dog sampling sites (red dots) where ectoparasites were collected. The inset box provides the geographic coordinates of the city centre and a representative sampling site. A north arrow and scale bar are included.

This research was conducted on dogs in Isfahan County, which is a large, relatively hilly area located in the central part of the Iranian plateau. The region's distinct geographical characteristics, combined with a hot and arid climate, agricultural practices and the presence of numerous suitable hosts, make Isfahan particularly vulnerable to different types of parasitic infections (Ayatollahi et al. 2022)(Figure 1).

### 2.2 Parasitological Procedures

For this research, a total of 138 dogs, comprising 57 stray dogs, 38 herd dogs and 43 owned dogs, were examined from July 2023 to July 2024 for the presence of ectoparasites in Isfahan County, Iran. Dogs were selected using stratified random sampling across urban, peri‐urban and rural sites in Isfahan County, with proportional allocation to owned, stray and herd categories. Sample size was calculated assuming 40% expected prevalence, 95% confidence and 5% precision, yielding a minimum of 369 dogs; however, due to logistical constraints, 138 dogs were examined. Their sex and age were documented, with age estimated using a dental formula. The animals in the study were categorized into two age groups (≤3 and >3 years old). Each dog was thoroughly examined for ectoparasites, which were removed using forceps, combing, or brushing and preserved in 70% ethanol. The preferred locations for the parasites (ears, head, back and flanks, chest and abdomen, limbs and tail) were noted.

Areas of the skin exhibiting dermatologic lesions were scraped deeply with an oily scalpel blade until capillary bleeding was observed. Deep otic swab samples were collected from all dogs to check for ear mites. Skin scrapings and ear swabs were then placed in 10% potassium hydroxide and gently heated for 30 min. Following this, the mixture was centrifuged and the sediment was examined microscopically for mites.

The species of ectoparasites were identified using a stereomicroscope based on established identification keys (Wall and Shearer 2008).

### 2.3 Statistical Analysis

Data were analysed using the Statistical Package for the Social Sciences software (version 26). Descriptive statistics were presented as frequencies and percentages. The associations between ectoparasite infestation (presence/absence) and categorical variables (sex, age group and dog type) were evaluated using the Chi‐square test or Fisher's exact test when expected frequencies were less than 5. Prevalence estimates are reported with 95% confidence intervals. The Mann–Whitney U test was applied only for comparing the median number of parasites recovered between two independent groups (e.g., male vs. female) when the data were non‐normally distributed. A two‐tailed *p*‐value < 0.05 was considered statistically significant. Due to the limited number of mixed infestations, multivariable logistic regression was not performed. Analyses were therefore restricted to univariate comparisons.

## 3 Results

### 3.1 Prevalence of Ectoparasite Infestation

In this study, of 138 dogs examined, 54 (39.13%; 95% CI: 31.39%–47.46%) dogs were infested with external parasites in Isfahan County. The characteristics of the infested dogs are summarized in Table 1. Statistical analysis revealed significant differences in the prevalence of ectoparasite infestation based on dog type, sex and age (*p* < 0.001 for all). Herd dogs showed the highest infestation rate (76.31%, 29/38), followed by stray dogs (31.57%, 18/57) and owned dogs (16.27%, 7/43). Male dogs had a higher infestation rate (57.37%, 35/61) compared to females (24.67%, 19/77). Infestation was more prevalent in dogs aged >3 years (59.18%, 29/49) than in those ≤3 years (28.08%, 25/89) (Table 1).

**TABLE 1:** Prevalence of ectoparasite infestation by sex, age and dog type (*n* = 138), with 95% confidence intervals.

Characteristic	Category	No. examined	No. infested (%)	95% CI	*p*‐value
Sex	Male	61	35 (57.37)	44.9–69.0	<0.001
	Female	77	19 (24.67)	15.9–35.9	
Age	>3 years	49	29 (59.18)	45.0–72.0	<0.001
	≤3 years	89	25 (28.08)	19.5–38.5	
Dog type	Owned	43	7 (16.27)	7.3–30.4	<0.001
	Stray	57	18 (31.57)	20.4–45.2	
	Herd	38	29 (76.31)	60.3–87.4	

Among the 54 infested dogs, 26 (48.14%; 95% CI: 35.39%–61.15%) were infested with ticks, 15 (27.77%; 95% CI: 17.62%–40.89%) with mites, 9 (16.66%; 95% CI: 9.02%–28.74%) with fleas and 4 (7.40%; 95% CI: 2.92%–17.55%) with lice. The prevalence of ectoparasites in dogs from Isfahan County based on age, sex and dog type is presented in Table 2.

**TABLE 2:** Distribution of ectoparasite‐infested dogs by sex, age group and dog type (*n* = 54 infested dogs).

Ectoparasites	Sex		Age		Dog type		
	Male	Female	≤3	>3	Owned	Stray	Herd
**Tick**	19	7	10	16	0	9	17
**Mite**	10	5	5	10	5	4	6
**Lice**	3	1	3	1	0	2	2
**Flea**	3	6	7	2	2	3	4
**Total**	35	19	25	29	7	18	29

The results of the seasonal prevalence of ectoparasite infestation in dogs from Isfahan County are presented in Table 3. According to the findings, the highest infestation rates with fleas and ticks were observed in spring (*p* < 0.05) (Table 3).

**TABLE 3:** Seasonal distribution of ectoparasite infestation in dogs (*n* = 54 infested dogs).

Identified ectoparasites in dogs (%)	Season				Total (heads)
	Spring	Summer	Autumn	Winter	
**Tick**	18 (69.23)	6 (23.07)	1 (3.84)	1 (3.84)	26
**Mite**	13 (86.66)	2 (13.33)	0	0	15
**Lice**	0	0	0	4 (100)	4
**Flea**	0	0	2 (22.22)	7 (77.7)	9

Ectoparasites were collected from various body regions of the examined dogs, including the ears, head, back and flanks, chest and abdomen, limbs and tail. The preferred attachment sites differed by parasite type and are summarized in Table 4.

**TABLE 4:** Preferred body‐site attachment of ectoparasites (percentages represent the distribution of collected parasites, not the proportion of infested animals).

Identified ectoparasites in dogs (%)	Body distribution (%)				
	Ear	Chest and abdomen	Limbs and tail	Back and flanks	Head
**Tick**	56.67	7.14	4.12	15.43	16.64
**Mite**	2.14	12.39	65.24	16.78	3.45
**Flea**	1.8	12.51	29.6	46.74	9.35
**Lice**	1.21	2.13	0	54.12	42.54

Concurrent infestation with more than one ectoparasite group was observed in 5 dogs (9.26%). A summary of single and mixed infestations is presented in Table 5.

**TABLE 5:** Frequency of single and mixed ectoparasite infestations in dogs (*n* = 54 infested dogs).

Type of infestation	No. of dogs	% of infested dogs
Single infestation	49	90.74
Mixed infestation	5	9.26
Total infested	54	100

### 3.2 Hard Tick Infestation

A total of 149 adult ixodid ticks were found on 26 out of 54 dogs examined, resulting in a prevalence of 48.14%, which included 17 herding dogs and 9 strays. The hard ticks identified were from the genera *Hyalomma* and *Rhipicephalus*, consisting of three species*: Rhipicephalus sanguineus* (69.28%), *Rhipicephalus bursa* (29.45%) and *Hyalomma marginatum* (1.27%). The highest level of tick infestation was recorded in the spring, with most ticks being removed from the dogs' ears.

### 3.3 Mite Infestation

In this study, 15 dogs (27.77%) were found to be infested with mites, including 6 herding dogs, 4 stray dogs and 5 owned dogs. The collected mites included *Sarcoptes scabiei canis* and *Demodex canis*. Concurrent infestations with ectoparasites were noted in 5 dogs, where four exhibited a co‐infestation of *Rhipicephalus sanguineus* and *Demodex canis* and one had both *Sarcoptes scabiei* mite and *Rhipicephalus bursa* tick present at the same time.

### 3.4 Flea and Lice Infestation

This research identified a total of 81 fleas, including *Ctenocephalides canis* and *Pulex irritans*, from 9 dogs (16.66%), which comprised 4 herding dogs, 3 strays and 2 owned dogs displaying signs of itching. The majority of fleas were found on the back regions of the affected dogs. Additionally, 49 lice of *Trichodectes canis* were collected from 4 dogs (7.40%), which included 2 herding dogs and 2 strays, during the winter season.

## 4 Discussion

Diseases resulting from external parasites, such as fleas and ticks, are prevalent in dogs and can cause significant health problems. With more than one million species of arthropods making up over 80% of all documented animal species, their impact on ecosystems and disease spread is indisputable. Environmental factors and interactions between species play crucial roles in the occurrence of parasitic infections in dogs (Pestehchian et al. 2012).

The current study found that the overall prevalence of ectoparasite infestations among 138 dogs in Isfahan County, Iran, was 39.13%, with ticks being the most common (48.14%), followed by mites (27.77%), fleas (16.66%) and lice (7.40%). This rate is considered moderate when compared to other regional and international research, underscoring the ongoing difficulty in controlling ectoparasites within dog populations, especially in regions that employ mixed dog management methods. These results correspond with the endemic characteristics of ectoparasites in tropical and subtropical regions, where environmental conditions promote parasite survival and dissemination (Ebrahimzade et al. 2016; Abdulkareem et al. 2019).

The prevalence of ectoparasite infestation observed in the present study (39.13%) was slightly lower than that reported in northwestern Iran (41.30%; Shirzadfar et al. 2025), but higher than the 21.52% reported in Mashhad (Minabaji et al. 2020). A markedly higher prevalence (82.8%) was documented among stray dogs in northern and Central Iran (Mazandaran, Gilan and Qazvin provinces) (Ebrahimzade et al. 2016). On an international scale, our findings were lower than those reported in Kwara State, Nigeria (81.4% among owned dogs; Abdulkareem et al. 2019) and Morogoro, Tanzania (71.7% among dogs with a history of regular dipping treatment) (Mwangengwa and Rhanga 2023). These variations can be attributed to differences in study populations (stray vs. owned dogs), sampling methods, climatic and environmental conditions and the effectiveness of ectoparasite control programs. In Isfahan, the moderate prevalence suggests that current veterinary and management practices provide only partial protection, as reinfestation remains a challenge. This is consistent with observations in Tanzania, where regular acaricide dipping failed to completely eliminate infestations, likely due to persistent environmental sources of parasites (Mwangengwa and Rhanga 2023).

Marked variations in infestation rates were detected based on the type of dog, with herd dogs exhibiting the highest prevalence (76.31%), followed by strays (31.57%) and owned dogs (16.27%). This trend highlights how outdoor exposure and inadequate hygiene contribute to ectoparasite transmission, as both herd and stray dogs often roam freely, increasing their contact with infested areas or other animals (Minabaji et al. 2020; Shirzadfar et al. 2025). The findings of studies examining prevalence patterns and host‐related factors associated with tick infestation in Tehran province (Abbasi 2025b) also support the significant differences in infestation rates among different host types (owned, stray and herd dogs) observed in the current study. Similar patterns were found in Mashhad, where outdoor dogs had higher infestation rates (Minabaji et al. 2020) and in Urmia, where strays and sheepdogs were more commonly affected (Shirzadfar et al. 2025). Conversely, owned dogs in Nigeria displayed a high prevalence despite being kept indoors, potentially due to insufficient knowledge among owners regarding parasite management (Abdulkareem et al. 2019). The study conducted in Tanzania further underscored environmental risks, noting a 15‐fold increase in infestation among dogs living on wooden or earthen floors, which can harbour parasite eggs and larvae (Mwangengwa and Rhanga 2023). These results imply that in Isfahan, focused interventions aimed at herd and stray dogs, such as enhancing housing sanitation, might lower prevalence rates.

Sex and age were identified as important factors associated with ectoparasite infestation, with male dogs (57.37%) and those older than 3 years (59.18%) showing elevated infestation rates. The increased prevalence in males corresponds with behavioural traits such as greater roaming and territorial behaviours, which heighten their exposure to parasites, as indicated in Urmia (Shirzadfar et al. 2025), Tehran (Jamshidi et al. 2012) and South Korea (Chee et al. 2008). However, this is in contrast to research conducted in northern Iran, where no correlation with sex was found (Ebrahimzade et al. 2016) and in Mashhad, where sex was relevant but exhibited different patterns (Minabaji et al. 2020).

The increased prevalence in older dogs is likely due to cumulative exposure over time, longer periods of outdoor activity and possible age‐related decline in immune competence. This finding is consistent with several studies conducted in Iran and other regions (Shoorijeh et al. 2008; Abdulkareem et al. 2019; Shirzadfar et al. 2025). However, it contrasts with some previous reports that found higher infestation rates in younger dogs (Mosallanejad et al. 2012). These variations may be attributed to differences in study populations, management practices and environmental conditions. Age‐specific control strategies should therefore be considered in Isfahan, with particular attention to older dogs.

Seasonal changes were significant, with the highest infestations of ticks and fleas occurring in spring. This pattern aligns with warmer temperatures that favour parasite reproduction and activity (Minabaji et al. 2020; Shirzadfar et al. 2025). Similarly, studies on livestock ticks in Tehran province have demonstrated strong associations between climatic variations and tick abundance (Abbasi 2025a), further supporting the important role of environmental factors in the epidemiology of ectoparasites in Central Iran. In Mashhad, peak infestation rates were also observed in spring and summer (Minabaji et al. 2020), whereas surveys in northern Iran reported higher flea prevalence during fall and winter, likely due to regional climatic differences (Ebrahimzade et al. 2016).

Research from Nigeria and Tanzania did not highlight seasonality but acknowledged continuous risks in tropical environments (Abdulkareem et al. 2019; Mwangengwa and Rhanga 2023). In Isfahan, lice infestations peaked during winter, corresponding with cooler temperatures that encourage closer contact among dogs (Shirzadfar et al. 2025).

In Isfahan, ectoparasite body‐site preferences showed ticks favoured the ears, mites were found on the limbs, fleas on the back and flanks and lice on the head, reflecting findings from other studies. In Mashhad, the neck and head were common sites for various parasites (Minabaji et al. 2020), and a similar trend was noted in Pakistan where the neck and head were also the main areas for ectoparasite infestation (Memon et al. 2018). Nigerian studies revealed comparable distribution patterns, with *Rhipicephalus sanguineus* preferring the ears and abdomen (Abdulkareem et al. 2019). Such preferences are valuable for guiding targeted examinations and treatments.

The ectoparasite species identified in Isfahan included *Rhipicephalus sanguineus* (69.28%), *Rhipicephalus bursa* (29.45%) and *Hyalomma marginatum* (1.27%) among ticks; *Sarcoptes scabiei* and *Demodex canis* among mites; *Ctenocephalides canis* and *Pulex irritans* among fleas; and *Trichodectes canis* for lice, which align with findings from similar studies. Ticks like *Rhipicephalus species* were also found to be predominant in various regions of Iran, such as Urmia (Shirzadfar et al. 2025), Ghilanegharb (Mirani et al. 2017), Shiraz (Shoorijeh et al. 2008), Ilam (Bahrami and Delpisheh 2010b), Midwest (Abbasi 2024) and northern Iran (Ebrahimzade et al. 2016). Fleas (*Ctenocephalides canis* and *Pulex irritans*) correspond with reports from Tanzania (Mwangengwa and Rhanga 2023) and other studies in Iran (Ebrahimzade et al. 2016; Minabaji et al. 2020). The mite and lice species remain consistent across research, highlighting their zoonotic potential (Mwangengwa and Rhanga 2023; Shirzadfar et al. 2025).

Several of the ectoparasites identified in this study are known vectors or agents of zoonotic diseases. Although the current study did not examine pathogen carriage or transmission, the relatively high prevalence of these ectoparasites in dogs that live in close proximity to humans suggests a potential public health risk. Further investigations are warranted to determine the role of these parasites in the transmission of zoonotic pathogens in the region.

This study has some limitations that should be considered when interpreting the results. First, due to the moderate sample size (*n* = 138) and the small number of dogs with mixed infestations (*n* = 5), we were unable to perform multivariable logistic regression analysis. Consequently, the associations identified between ectoparasite infestation and variables such as sex, age and dog type may be influenced by confounding factors. Future studies with larger sample sizes are recommended to confirm independent risk factors and calculate adjusted odds ratios. Second, although dogs were selected using stratified random sampling, the final sample size was lower than the calculated minimum required for 5% precision, which may limit the generalizability of the findings. Despite these limitations, the study provides valuable regional epidemiological data on canine ectoparasites in Central Iran.

## 5 Conclusion

In summary, the findings underscore the varying prevalence of ectoparasites among dogs in Isfahan, which is affected by factors such as the type of dog, sex, age, seasonal variations and specific body regions. Therefore, integrated control programs including regular ectoparasite treatment and improved hygiene are recommended to reduce both animal health problems and potential zoonotic risks.

## Author Contributions

**Farnaz Malekifard** and **Bijan Esmaeilnejad**, contributed to conception, design, data collection, statistical analysis and drafting of the manuscript. **Nafas Rafiyan**, **Farnaz Malekifard** and **Bijan Esmaeilnejad**, contributed to conception, design, supervision of the study and drafting of the manuscript. All authors approved the final version for submission.

## Funding

The authors have nothing to report.

## Conflicts of Interest

The authors declare no conflicts of interest.

## Ethics Approval Statement

Ethical considerations of the study was approved by Animal Ethics Committee in Urmia University, Urmia, Iran (IR‐UU‐AEC‐3/74) and conducted under the regulations of this committee.

## Data Availability

The data that support the findings of this study are available on request from the corresponding author. The data are not publicly available due to privacy or ethical restrictions.

## References

[vms371191-bib-0001] Abbasi, E. 2024. Biodiversity, geographical distribution, and faunal study of tick populations infesting livestock in an Elevated County of Midwest Iran. *Available at SSRN 4701483*. 10.2139/ssrn.4701483.

[vms371191-bib-0002] Abbasi, E. 2025a. “Assessing the Influence of Seasonal and Climatic Variations on Livestock Tick Incidence in Tehran Province, Iran: Cross‐Sectional Study.” JMIRx Bio 3, no. 1: e69542.

[vms371191-bib-0003] Abbasi, E. 2025b. “Prevalence and Identification of Livestock Tick by Sex Ratio and Host in Tehran Province.” Veterinary Medicine and Science 11, no. 6: e70702. 41251265 10.1002/vms3.70702 PMC12624458

[vms371191-bib-0004] Abdulkareem, B. O. , A. L. Christy , and U. U. Samuel . 2019. “Prevalence of Ectoparasite Infestations in Owned Dogs in Kwara State, Nigeria.” Parasite Epidemiology and Control 4: e00079. 30662964 10.1016/j.parepi.2018.e00079 PMC6324013

[vms371191-bib-0005] Ayatollahi, J. , M. Jeyhaninejad , S. A. Mousavi , M. Hamidfar , and S. Hossein . 2022. “The Frequency of Intestinal Parasites in the Samples of Alzahra Laboratory of Isfahan (2017‐2019).” Journal of Medical Research 8, no. 2: 56–58.

[vms371191-bib-0006] Bahrami, A. M. , and A. Delpisheh . 2010. “Common Ectoparasite Species of Domestic Dogs in Western Iran.” World Applied Sciences Journal 8, no. 10: 1277–1281.

[vms371191-bib-0007] Bahrami, A. M. , A. Doosti , and S. Ahmady_Asbchin . 2012. “Cat and Dogs Ectoparasite Infestations in Iran and Iraq Boarder Line Area.” World Applied Sciences Journal 18, no. 7: 884–889.

[vms371191-bib-0008] Beresford‐Jones, W. P. 1981. “Prevalence of Fleas on Dogs and Cats in an Area of Central London.” Journal of Small Animal Practice 22, no. 1: 27–29. 7193779 10.1111/j.1748-5827.1981.tb01388.x

[vms371191-bib-0009] Chee, J.‐H. , J.‐K. Kwon , H.‐S. Cho , et al. 2008. “A Survey of Ectoparasite Infestations in Stray Dogs of Gwang‐ju City, Republic of Korea.” Korean Journal of Parasitology 46, no. 1: 23. 18344673 10.3347/kjp.2008.46.1.23 PMC2526298

[vms371191-bib-0010] Dantas‐Torres, F. , and D. Otranto . 2014. “Dogs, Cats, Parasites, and Humans in Brazil: Opening the Black Box.” Parasites & Vectors 7, no. 1: 22. 24423244 10.1186/1756-3305-7-22 PMC3914713

[vms371191-bib-0011] Durden, L. A. , T. N. Judy , J. E. Martin , and L. S. Spedding . 2005. “Fleas Parasitizing Domestic Dogs in Georgia, USA: Species Composition and Seasonal Abundance.” Veterinary Parasitology 130, no. 1–2: 157–162. 15893082 10.1016/j.vetpar.2005.03.016

[vms371191-bib-0012] Ebrahimzade, E. , R. Fattahi , and M. B. Ahoo . 2016. “Ectoparasites of Stray Dogs in Mazandaran, Gilan and Qazvin Provinces, North and Center of Iran.” Journal of Arthropod‐Borne Diseases 10, no. 3: 364. 27308294 PMC4906742

[vms371191-bib-0013] González, A. , D. del C Castro , and S. González . 2004. “Ectoparasitic Species From *Canis familiaris* (Linné) in Buenos Aires Province, Argentina.” Veterinary Parasitology 120, no. 1–2: 123–129. 15019149 10.1016/j.vetpar.2003.12.001

[vms371191-bib-0014] Gracia, M. J. , C. Calvete , R. Estrada , J. A. Castillo , M. A. Peribanez , and J. Lucientes . 2008. “Fleas Parasitizing Domestic Dogs in Spain.” Veterinary Parasitology 151, no. 2–4: 312–319. 18031934 10.1016/j.vetpar.2007.10.006

[vms371191-bib-0015] Jamshidi, S. , N. Maazi , S. Ranjbar‐Bahadori , M. Rezaei , P. Morakabsaz , and M. Hosseininejad . 2012. “A Survey of Ectoparasite Infestation in Dogs in Tehran, Iran.” Revista Brasileira de Parasitologia Veterinaria 21: 326–329. 23070452 10.1590/s1984-29612012000300030

[vms371191-bib-0016] Memon, M.‐R. , J. A. Baloch , and A. Arjio , et al. 2018. “Studies on the Prevalence of Ectoparasites in Owned Dogs and Major Risk Infestation to Human Health in Karachi, Sindh Pakistan.” Pakistan Journal of Parasitology 65: 19–29.

[vms371191-bib-0017] Minabaji, A. , A. Moshaverinia , and J. Khoshnegah . 2020. “Frequency of Ectoparasite Infestation in Dogs in Mashhad, Northeast Iran.” Journal of Veterinary Research/Majallah‐i Taḥqīqāt‐i Dāmpizishkī University 75, no. 3: 459–466.

[vms371191-bib-0018] Mirani, F. , M. Yakhchali , and S. Naem . 2017. “A Study on Ectoparasites Fauna of Dogs in Suburbs of Ghilanegharb, Kermanshah Province, Iran.” Journal of Veterinary Research 72, no. 1: 364–369.

[vms371191-bib-0019] Mosallanejad, B. , A. R. Alborzi , and N. Katvandi . 2012. “A Survey on Ectoparasite Infestations in Companion Dogs of Ahvaz District, South‐West of Iran.” Journal of Arthropod‐Borne Diseases 6, no. 1: 70. 23293781 PMC3528164

[vms371191-bib-0020] Mwangengwa, L. M. , and S. Rhanga . 2023. “Assessment of the Prevalence and Risk Factors of Ectoparasite Infestation in Dogs With Regular Dipping History in Morogoro Urban and Peri‐Urban in Tanzania.” Journal of Veterinary Science 4, no. 1: 1019.

[vms371191-bib-0021] Nuchjangreed, C. , and W. Somprasong . 2007. “Ectoparasite Species Found on Domestic Dogs From Pattaya District, Chon Buri Province, Thailand.” Southeast Asian Journal of Tropical Medicine and Public Health 38, no. 1: 203.

[vms371191-bib-0022] Pestehchian, N. , A. Rasouli , and H. A. Yoosefi . 2012. “Distribution of Intestinal Worms Among Stray Dogs in Isfahan, Iran.” Journal of Isfahan Medical School 29, no. 172: 2827–2833.

[vms371191-bib-0023] Scott, D. W. , W. H. Miller , C. E. Griffin , and G. H. Muller . 2001. Canine scabies. Muller & Kirk's Small Animal Dermatology. 6th ed, 476–485. WB Saunders.

[vms371191-bib-0024] Shirzadfar, S. , F. Malekifard , and B. Esmaeil Nejad . 2025. “Prevalence of Ectoparasite Infestation in Dogs in Urmia Suburb, Iran: A Cross‐Sectional Study.” Iranian Journal of Animal Science 56, no. 3: 617–626.

[vms371191-bib-0025] Shoorijeh, S. J. , A. R. Ghasrodashti , A. Tamadon , N. Moghaddar , and M. A. Behzadi . 2008. “Seasonal Frequency of Ectoparasite Infestation in Dogs From Shiraz, Southern Iran.” Turkish Journal of Veterinary & Animal Sciences 32, no. 4: 309–313.

[vms371191-bib-0026] Šlapeta, J. , J. King , D. McDonell , et al. 2011. “The Cat Flea (Ctenocephalides f. felis) Is the Dominant Flea on Domestic Dogs and Cats in Australian Veterinary Practices.” Veterinary Parasitology 180, no. 3–4: 383–388. 21515000 10.1016/j.vetpar.2011.03.035

[vms371191-bib-0027] Thompson, R. C. A. , B. P. Meloni , R. M. Hopkins , P. Deplazes , and J. A. Reynoldson . 1993. “Observations on the Endo‐and Ectoparasites Affecting Dogs and Cats in Aboriginal Communities in the North‐West of Western Australia.” >Australian Veterinary Journal 70, no. 7: 268–270. 8368974 10.1111/j.1751-0813.1993.tb08050.x

[vms371191-bib-0028] Wall, R. L. , and D. Shearer . 2008. Veterinary Ectoparasites: Biology, Pathology and Control. John Wiley & Sons.

[vms371191-bib-0029] Xhaxhiu, D. , I. Kusi , D. Rapti , et al. 2009. “Ectoparasites of Dogs and Cats in Albania.” Parasitology Research 105, no. 6: 1577–1587. 19690887 10.1007/s00436-009-1591-x

